# Exploring factors influencing initiation, implementation and discontinuation of medications in adults with ADHD

**DOI:** 10.1111/hex.13031

**Published:** 2020-02-07

**Authors:** Muhammad Umair Khan, Parisa Aslani

**Affiliations:** ^1^ The University of Sydney School of Pharmacy Faculty of Medicine and Health The University of Sydney Camperdown NSW Australia

**Keywords:** ADHD, adherence, adults, discontinuation, focus groups, implementation, initiation, qualitative

## Abstract

**Background:**

Adherence to ADHD medication is a complex phenomenon as the decision to adhere is influenced by a range of factors. To design tailored interventions to promote adherence, it is important to understand the factors that influence adherence in the context of its three phases: initiation, implementation and discontinuation.

**Objective:**

The objective of this study was to explore the phase‐specific factors that influence adherence to medication in adults who have a diagnosis of ADHD.

**Methods:**

Three focus groups (FGs) were conducted with twenty adults with ADHD in different metropolitan areas of Sydney, Australia. FGs were transcribed verbatim and thematically analysed.

**Results:**

Participants’ decision to initiate medication (the initiation phase) was influenced by their perceived needs (desire to improve academic and social functioning) and concerns (fear of side‐effects) about medication following a similar process as defined by the Necessity‐Concerns Framework (NCF). The balance between benefits of medication (needs) and side‐effects (concerns) continued to determine participants’ daily medication‐taking (the implementation phase) and persistence (or discontinuation) with their medication. Forgetfulness and stigma were reported as concerns negatively impacting the implementation phase, while medication cost and dependence influenced the discontinuation phase of adherence.

**Conclusions:**

Adults’ decision to initiate, continue or discontinue medication is influenced by a range of factors; some are unique to each phase while some are common across the phases. Participants balanced the needs for the medication against their concerns in determining whether to adhere to medication at each phase. It appears that the NCF has applicability when decision making about medication is explored at the three phases of adherence.

## 1 BACKGROUND

Attention‐deficit hyperactivity disorder (ADHD) is a neurodevelopmental disorder marked by persistent inattention and/or hyperactivity‐impulsivity.^1^ Although ADHD is predominantly a childhood disorder, it often continues into adulthood, with reports that approximately two‐thirds of children with ADHD continue to have signs and symptoms of ADHD into adulthood, either unabated or with partial improvement.^1^ The reported prevalence in adults varies from 1.2% to 7.3%^2^ and may even be higher as the disorder may be underdiagnosed in adults.^3^, ^4^

Evidence suggests that about 90% of adults with ADHD have at least one comorbid psychiatric disorder.^5^ Moreover, these adults are at increased risk of alcohol dependency, drug misuse, car accidents and suicide.^6^, ^7^ Lack of focus, disorganization, forgetfulness, impulsivity, time management problems and relationship issues are all symptoms of ADHD in adults.^8^ These symptoms have been associated with academic, occupational and social difficulties,^9^, ^10^ warranting the need for ADHD management. Despite the availability of behavioural management, pharmacotherapy plays an important role in the management of ADHD.^11^

Attention‐deficit hyperactivity disorder medications reduce the symptoms and improve daily functioning and quality of life of adults with ADHD.^12^ However, adherence is a key factor associated with the effectiveness of ADHD medication and treatment outcomes. Evidence suggests that adherence to medication among adults with ADHD is sub‐optimal. A study reported that 88% of adults with ADHD were inconsistent in taking their medication,^13^ and 41.2% of adults were non‐adherent to ADHD medication.^10^ Furthermore, adult patients with lower adherence to medication (less than 80%) had considerably higher ADHD severity than those with higher adherence (more than 80%).^14^

Medication adherence is influenced by a number of factors, which can change as the patient progresses through the different phases of medication‐taking.^15^ To better understand the complexity of this journey, Vrijens et al^16^ proposed the Ascertaining Barriers to Compliance (ABC) taxonomy. The ABC taxonomy provides a more transparent and rigorous approach to examine adherence in the context of its three phases: initiation, implementation and discontinuation. The initiation phase was defined as consumption of the first dose of a prescribed medication. The implementation phase was defined as the extent to which patients adhere to the prescribed dosing regimen from initiation until the last dose of the medication. The discontinuation phase was defined as the cessation of the prescribed medication for any given reason.^16^ Furthermore, the ABC taxonomy proposed to identify all possible causes of non‐adherence, including intentional and unintentional non‐adherence, to better understand the complex and dynamic nature of adherence.

Patients’ beliefs about medication are an important factor that determine patients’ adherence to medication.^17^ The Necessity‐Concerns Framework (NCF) argues that patients’ beliefs about medication adherence are based on their perceived need (necessity) and concerns about medication.^17^ Patients are more likely to adhere if their perceived needs for the medication outweigh their concerns and vice versa. The NCF accounts for both intentional and unintentional non‐adherence.^18^ This framework has been successfully applied across a range of medical conditions^18^; however, little is known about its use in adults with ADHD.

Despite the evidence for poor medication adherence in adults with ADHD, only a small number of studies have examined the factors influencing medication adherence.^10^, ^12^, ^19^, ^20^, ^21^ These studies have reported psychiatric comorbidity, treatment settings (specialist vs general physician), forgetfulness, gender, education, age and family history as the main factors that influence medication adherence.^10^, ^12^, ^19^, ^20^, ^21^ However, none of these studies have examined adherence in view of its dynamic nature, or in the context of its influence on the three phases of adherence. Evidence from different disease populations suggests that different factors can influence different phases of adherence.^22^, ^23^, ^24^ The objective of this study was, therefore, to explore the factors that influence adherence to ADHD medication in the context of the three phases of adherence (initiation, implementation and discontinuation) in adults with ADHD.

## 2 METHODS

### 2.1 Study design

Given the exploratory nature of this study, a qualitative approach was used.^25^ Focus groups (FGs) were preferred as they provide an opportunity to generate richer data through interactions and discussions between participants compared to individual interviews.^26^ The reporting structure of this study was guided by the consolidated criteria for reporting qualitative research (COREQ)^27^ (Appendix A).

### 2.2 Study participants

After receiving approval from the institution's Human Research Ethics Committee (Project no. 2018/271), participants were recruited through a market research company. Convenience sampling was used to recruit participants from different geographical locations (centre, south and west) of Sydney to ensure diversity in the participants’ socio‐economic characteristics. Each participant was reimbursed AUD80 for their time and travel expense. FGs were conducted in English. Participants were recruited if they were aged between 18 and 65 years, diagnosed with ADHD, prescribed ADHD medication and able to participate without the need for an interpreter. Twenty adults with ADHD participated in three FGs (Table 1). Two FGs had seven participants each while one FG had six participants. Most participants initiated medication during adulthood (n = 8) while some initiated in their childhood (n = 6) and some in their adolescence (n = 6).

**Table 1:** Summary of participants’ demographic information (n = 20)

Demographics	Median (IQR)
Age (in years)	34 (11.5)
**Number of participants**	
Time of diagnosis (age range)	
Childhood (4 −12 years)	7
Adolescence (13 ‐ 17 years)	5
Adulthood (18 years or above)	8
Gender	
Male	11
Female	9
Highest education	
Secondary (Year 7 to 12)	10
TAFE^2^ (Vocational education and training)	3
University (University education after year 12)	7
Joint family income (annual, AUD $)^3^	
18 200 or less	2
18 201‐37 000	3
37 001‐90 000	7
90 001‐180 000	5
180 001 or over	1
Marital status	
Single	12
Married	2
Defacto^4^	4
Divorced	2
Employment status^5^	
Working full‐time	10
Working part‐time	4
Student	6
Not working	3
Current medication^5^	
Methylphenidate – short‐acting	10
Methylphenidate – long‐acting	1
Methylphenidate – extended release	1
Dexamfetamine	3
Atomoxetine	2
None	5

Technical and Further Education (TAFE) awards Australian Qualifications Framework (AQF) qualifications accredited in the Vocational Education and Training (VET) sector.

Two participants did not provide a response.

According to the Australian Family Law Act 1975, de facto is defined as a relationship between two people who are living together on a genuine domestic basis and are not legally married or related by family.

Some participants chose more than one response.

### 2.3 Focus group discussions

Prior to attending the FGs, potential participants were provided with the participant information statement (which included detailed information about the study, and other issues such as confidentiality, anonymity and voluntary nature of participation) and consent form. They were provided the same information at the beginning of the FGs to ensure that they had read the information and had the opportunity to ask questions about the study. Participants were asked to complete a brief demographic information form (Appendix B). All participants provided written consent prior to commencing the FG discussions.

FGs were conducted between August 2018 and January 2019 at venues intended for FGs. Efforts were made to provide an informal and relaxed but professional environment to ensure that participants felt comfortable. Two FGs were moderated by an experienced facilitator (PA). MUK acted as an observer and took field notes on the key points raised during the group discussions. One FG was moderated by MUK under the supervision of PA. To minimize moderator bias and ensure consistency between the FGs, a semi‐structured FG protocol was developed by reviewing previous studies^10^, ^12^, ^19^, ^20^, ^21^ in line with the study objectives (Appendix C). The format and style of the questions were in accordance with the criteria proposed by Krueger and Casey.^28^ The content of the questions was based on the ABC taxonomy.^16^ Each FG lasted approximately 60 minutes. All FGs were audio‐recorded with the permission of the participants and transcribed verbatim.

### 2.4 Data analyses

The data were analysed by MUK and PA using a framework for thematic analysis.^29^ The NVIVO 11 Pro computer software was used for data management. The process of data analysis started during the phase of data collection. After each session, MUK and PA discussed the themes that arose during the discussions. No new codes were generated from the 3rd FG discussions, and it was decided by the authors that data saturation had been reached.^30^ Thematic analysis was performed in six steps. In the first step, MUK read the FG transcripts and listened to the audio‐recordings numerous times to become familiar with the data. In the second step, the initial codes were generated by MUK through inductive coding. A latent approach was used for coding the transcripts to capture the conceptual and underlying meaning of the data. Each code was grouped under one of the three phases of adherence based on the ABC taxonomy. In the third step, the codes were merged to form broader themes within each of the three phases following a discussion between MUK and PA. A few themes overlapped between the implementation and discontinuation phase, which were combined under the two phases to avoid duplication. In the fourth step, the themes were reviewed by MUK and PA for further refinement in the context of the overall data set. The iterative process of rearranging codes and themes was reflexive,^31^ moving back and forth through the transcripts to search for common and recurrent themes, which were then named and finalized after a thorough discussion between the researchers in the fifth step of the analysis. Disagreements between the researchers were resolved by discussion until a consensus was reached. In the final step (manuscript preparation), the themes were carefully presented to ensure that the research objectives were answered in the context of the original data set. The NCF was used to identify participants’ needs for and concerns about medication at the three phases of adherence.

Similar to the concept of validity and reliability of qualitative study instruments, scholars emphasize establishing trustworthiness in qualitative research. In this study, trustworthiness of the findings was established during each step of the thematic analyses by following the strategies suggested by Nowell et al,^32^ and guided by the concept of trustworthiness presented by Lincoln and Guba.^33^ The means used to establish trustworthiness are presented in Table 2.

**Table 2:** Summary of strategies to establish trustworthiness

Phases of thematic analysis	Means of establishing trustworthiness
Familiarization with data	Prolong engagement with data Store raw data in well‐organized archives Keep records of all data field notes and transcripts
Initial coding	Use of a coding framework Audit trail of code generation
Searching for themes	Researcher triangulation Keep detailed notes about development and hierarchies of concepts and themes
Reviewing themes	Themes and subthemes vetted by team members
Defining and naming themes	Team consensus
Producing the report	Describing process of coding and analysis in sufficient detail Thick descriptions of context Team consensus

## 3 RESULTS

Numerous factors were reported that influenced participants’ perceived need (necessity beliefs) and their concerns about medication (Table 3). The findings are presented under two major themes in accordance with the NCF. Within each theme, factors have been categorized based on their influence on the three phases of adherence. The two major themes are the following:

1. participants’ need for medication
2. participants’ concerns about medication

**Table 3:** Participant's perceived needs and concerns about medication at the three phases of adherence

Perceived needs	Concerns
Initiation	
Desire to improve ADHD outcomes Trust in physician	Negative beliefs
Implementation	
Benefits of medication^6^ Fear of life without medication^6^ Limited ability to self‐control^6^	Forgetfulness Medication stigma Medication side‐effect^6^
Discontinuation	
Benefits of medication^6^ Fear of life without medication^6^ Limited ability to self‐control^6^ Lack of alternative options	Medication side‐effect^6^ Dependency Cost

Factors which were common to the implementation and discontinuation phases.

### 3.1 Participants’ need for medication

#### 3.1.1 The initiation phase

At the initiation phase, participants’ perceived need for medication was influenced by two factors: challenges of dealing with ADHD and trust in physician. Both factors increased the necessity beliefs of participants that encouraged them to commence therapy.

##### Desire to improve ADHD outcomes

The persistent academic, occupational and social challenges of living a life with ADHD increased the necessity beliefs of the participants to take medication. For some participants, these challenges started from childhood with problems at school due to inattention and aggressive behaviour. During adolescence, lack of concentration, aggressive behaviour and the strong feeling of rebellion were the common challenges. During adulthood, one of the most common issues was poor interpersonal communication. Participants reported that they got very loud while talking and their voice (tone and pitch) changed during a conversation without realizing that they were sounding different. This consequently impacted their social interactions. Participants felt there was no empathy for people suffering from ADHD. This was not only limited to the general public but also to family members and work colleagues. Mind wandering was another worrying issue that participants had to deal with, which they felt impacted their ability to perform daily chores, and often put them in an embarrassing situation, especially during conversations. Participants reported that these challenges were draining their energy daily, and they were looking for help. They wanted to improve their behaviour and their academic and social performance. The desire to improve outcomes enhanced their need for medication.

Just exhausted of trying to deal with it (ADHD). I needed to go to the doctor to get it diagnosed and take medication. That was the main drive. (FG1, P1)

(I wanted to start medication) because of my behaviour, to see if it helps. (FG1, P7)

##### Trust in physician

Trust in physician was another factor that positively contributed to participants’ perceived need for medication. Those who initiated their medication during adulthood (n = 8) reported that their trust in the physician helped them in deciding to initiate medication as they believed that the physician would not prescribe any medication that would harm them.

Yes, it was fine with me (to start medication). I trusted he wouldn’t prescribe a drug that is bad for me. (FG1, P5)

#### 3.1.2 The implementation and discontinuation phase

The factors influencing participants’ need for medication at the implementation and discontinuation phase have been presented together as there were a few factors that overlapped between the two phases, while some factors were unique to each phase.

##### Benefits of medication

Once the medication was initiated, some participants experienced the benefits of the medication and it reinforced their beliefs about the need for medication. Participants who benefited were more inclined to continue taking their medication. However, participants differed in what they regarded as beneficial outcomes of medication‐taking. For example, improved concentration and more control over ADHD and their life were two important beneficial outcomes reported.

It improves concentration quite exponentially and feels like more control. I can be more organised. (FG1, P1)

Some participants mentioned that they take medication as it makes them more normal, settles their anxiety and helps them function better.

The main reason I take (medication) because that balances me and my brain. It seems like that I am normal, seems like I am talking because sometimes I have problem talking. I find it hard to get it out whatever I got to say. (FG1. P7)

Some participants had their concerns about the medication; however, they mentioned that the benefits of taking medication outweighed their concerns and helped them continue taking medication.

I always do worry generally about taking medications. I am not a scientist, I don’t understand about every little aspect of ingredient that affects me. I suppose I do think about that, but it’s not anything that would take me off medication because it’s working for me (FG1, P1)

##### Fear of living a life without medication

At the implementation phase, participants were well aware of their life with and without medication. Those who were experiencing the benefits of medication did not want to go back to their pre‐medication life. The fear of living a life without medication positively contributed to their adherence. This factor was also prominent in the discontinuation phase where some participants reported the fear of aggressive behaviour and ‘bad thoughts’ as their reasons for not discontinuing. Further probing revealed that ‘bad thoughts’ were more about harming other people, such as co‐workers.

That one day when I tested it out (by stopping medication), I was having very bad thoughts, like really bad thoughts, and I was like this is isn’t going well (FG2, P4)

And as soon as you stop taking (medication), your behaviour changes dramatically that the people that you love the most, you are affecting them and then you decide well I have to stay on the medication otherwise I don’t know; the consequences are too bad (FG2, P5)

I am very scared to go back to what I was 4 years ago (without medication). I am very happy and this is very confirmed to me to stay on the medication (FG1, P1)

##### Self‐control

Participants’ inability to control their own behaviour without medication reinforced their need to adhere. Similarly, participants who were able to control their symptoms without their medications were more in favour of discontinuing their medication. A few participants mentioned that they wanted to discontinue, but they were unable to control their behaviour and went from being stable on medication to violent and aggressive without medication, which made them restart the medication.

I don’t like being off it. I am completely different, aggressive, and very abusive, you touch me I punch you; I am very on edge, very violent. One day I had a rope wrapped around my neck and my mum pulled it off and I practically smacked her with…this is not fun. (FG1, P4)

##### Lack of alternatives

Some participants believed that there were no other effective alternatives to medication. They mentioned that they might think of discontinuing medication if they find better alternatives; however, most felt that medication was the best treatment they could use to manage their symptoms. Some of the alternatives discussed during the FGs were good diet, exercise, doing less university, more sleep and the use of natural remedies to stabilize mood.

(I would discontinue medication) if there was an alternative, if you could come up with an alternative to taking the medication that has proven to work, that would have been great. (FG2, P2)

### 3.2 Participants’ concerns about medication

#### 3.2.1 The initiation phase

##### Negative beliefs about medication

Participants who initiated medication during adulthood, and a few who initiated during adolescence, expressed their perceived concerns about starting medication. Severe side‐effects and negative perceptions towards pharmaceutical companies mainly constituted their negative beliefs towards ADHD medication. Participants who had started medication during childhood did not report any specific personal concerns, but one participant recalled that despite having ADHD symptoms her parent did not want to initiate medication as the parent was not in agreement with treating a child with stimulants.

I was very distractible at school and always in trouble. I was a very sporty child, but yeah my mum wouldn’t medicate me. She didn’t like those stimulant‐based medications. (FG2, P2)

Pharmaceutical companies came into power and wanted to push their drugs and medicate the society. It’s called the disorder which kind of inspires people to medicate their children, and they don’t know what type of crap goes in. I wanted to find ways around it and use medication as a last resort (FG3, P5)

#### 3.2.2 The implementation and discontinuation phase

##### Forgetfulness

Once the medication was initiated, forgetfulness was one issue that concerned most participants about medication‐taking and resulted in unintentional non‐adherence. Occasionally, forgetfulness also led to confusion or doubts if they had taken medication at all. One participant mentioned that a change in sleeping patterns made her more forgetful while another participant recognized a change in eating patterns as a reason to forget his medication as he always took the medication with food. Overall, participants took measures to remind themselves to take their medication on time such as keeping their medication at the bedside or setting an alarm.

When I take tablet, I do tend to forget sometimes although I make an alarm to take them. I just stick to it (medication) next to my bed to make sure that I take them. (FG1, P6)

I tried to adhere because I wanted to take it when I was eating and that was the main problem because I always forget breakfast to eat or I eat very late like 2 or something. (FG1, P5)

I’m just lazy when it comes to timing. I wake up at 4 o clock and I am up until midnight may be that’s the reason (I forget). (FG3, P6)

##### Medication stigma

Stigma experienced by participants because of the medication they took, negatively impacted their adherence to their medication. Participants who took medication as a child or adolescent reported facing stigma mainly at school. As adults, participants discussed the stigma felt by people with ADHD, and specifically, the stigma they felt from people who knew they were on medication such as people at their workplace.

As you were saying about kids at school, the most common one I got was why are you taking tablets? Are you retarded? (FG1, P4)

I’ll be seen as a bad person to other people so it’s like the stigma attached to the medication (FG2, P5)

##### Medication side‐effects

Medication side‐effects were the most common concern among participants that influenced their decision to continue or discontinue taking medication. Headaches, loss of appetite, weight loss, insomnia, nausea, diarrhoea, back spasms, glare, depression and paranoia were common side‐effects reported by the participants.

It (medication) gave the problems pretty quickly. I started losing weight and eventually, I started to stay away from it. (FG1, P5)

I dreamt my mother was murdered in the middle of the night. She woke me up, and I was sitting beside her bed crying, and I said, you are dead. That was really horrible! (FG1, P4)

##### Dependency

A few participants discontinued taking their medication because of fear that they may become dependent on the medication, while some participants were trying to discontinue but reported that they felt anxious when they did not take ADHD medication. Overall, participants expressed their desire to discontinue medication to avoid complete dependence on medication.

I have been off this medication and tried other things. I have just found that I couldn’t do it, but I would really like to get off the medication. I don’t know how you wean off the drug. (FG2, P2)

##### Cost

A few participants had discontinued taking medication because of the associated cost, while a few were thinking of discontinuing due to the increasing cost of the medication. Interestingly, participants who had discontinued their medication wanted to be back on it but considered cost as a big factor.

They (Doctor) would say if (one medication) doesn’t work, try this, try that, and I am like hang on a minute, are you paying for these medications? These meds are $30 or $40, you don’t have to pay. I wish if we were helped with the cost. I’ll take what you want but I am not gonna pay you $30, $40 or $50 for the tablets…no way…just keep it. (FG1, P3)

I would like to go back on it, but it costs a lot of money to get back on it. (FG2, P7)

## 4 DISCUSSION

The findings of this study provide insight into the factors that influence the decision to adhere to medication in adults with ADHD. Adults reported medication‐taking as a journey that started from their negative experiences with ADHD including academic, occupational or social challenges, which encouraged them to initiate medication. These experiences formed the necessity beliefs that they need medication to improve ADHD outcomes and these potential outcomes encouraged them to initiate medication. However, intentional non‐adherence was also noticed at this phase as some participants had more doubts towards their perceived needs and had higher concerns about taking medication, such as fear of side‐effects. These findings suggest that adults’ decision to adhere to medication at the initiation phase is based on their perceived need versus concerns about medication. Adults are more likely to adhere if their perceived need for medication is higher than their concerns about medication. Similarly, perceived concerns, if exceed perceived needs, may result in delayed initiation or no initiation at all. This relationship needs to be quantified to better understand the extent of association between medication initiation and the necessity‐concerns beliefs. The decision to start medication has been linked with patients’ beliefs in other disease populations.^34^

It is important to note that participants who initiated medication at different times, that is as a child, adolescent or adult, reported relatively different needs for initiating medication. The primary reasons reported by adults for initiating medication during their childhood, adolescence and adulthood were education and learning, aggressive behaviour and interpersonal communication, respectively. These factors may highlight the predominant issues faced by the participants at the different stages of their life and may be reflective of their symptoms. For example, as a child diagnosed with ADHD, the parents may have been keen to commence therapy in order to help their children focus better at school and therefore improve their learning and education. However, during teenage years, aggressive behaviour may have been a predominant symptom with adverse impacts on social interactions and performance at school. A few adults who initiated medication during childhood or adolescence reported limited involvement in the decision making to start medication, suggesting a lack of communication between parents and children on the use of medication. Evidence shows that children with ADHD and their parents may have different opinions on the use of medication.^35^ Active communication is required to address these differences as the difference of opinions can negatively influence medication adherence and its outcomes.

Our findings show that a trusting relationship between physician and patient can help in strengthening the necessity beliefs of adults with ADHD against their concerns, which can help them in initiating medication. Since ADHD is less recognized in adults compared to children,^36^ there is limited understanding of the relationship between physicians and adult patients, the level of trust with each other and the level of treatment alliance between them. Evidence shows that a good relationship with a physician can successfully influence a person's decision to initiate medication.^37^, ^38^

Once initiated, the decision to continue medication was influenced by participants’ experiences with medication. The results show that participants constantly balance their positive experiences (benefits) against negative experiences (concerns) to decide on continuing medication. However, this decision is not straightforward for adults as they struggle to find the right balance and often trade‐off between benefits and concerns about medication, consistent with previous research.^39^ The positive experience is mainly influenced by the benefits of medication that improve their educational, occupational, social and family functioning while their negative experience is primarily related to their experience of having medication side‐effects and stigma. These findings suggest that adults whose positive experience with medication outweighs the negative experience are more likely to adhere to medication at the implementation phase. Adherence at the implementation phase could be improved by ensuring that the most appropriate medication and dosing regimen has been prescribed. For example, non‐adherence to medication due to stigma at the workplace could be addressed by giving the person a long‐acting medication for ADHD.

As the balance shifts more towards the negative experiences, that is, if the desired benefits have not been achieved or the frequency or intensity of side‐effects increases and are not worth the benefits, adults consider discontinuing medication. Our findings suggest that side‐effects are the primary reason for discontinuation. The trade‐off between the benefits and concerns about medication suggests that adults make a conscious decision to adhere or not adhere to medication based on their experiences, highlighting the dominance of intentional non‐adherence at the implementation and discontinuation phase. Addressing the perceptual barriers, such as effective management of side‐effects, can improve the overall experience of adults with medication, which can help in improving intentional non‐adherence.^40^, ^41^ Depending on the side‐effect, various strategies could be used such as dose titration, careful monitoring, switching to another medication, taking medication with food (for nausea), appropriate diet plan (for weight loss), drinking plenty of fluid (for headaches) and a change in medication timing (for insomnia).^42^, ^43^

This qualitative study confirms previous findings^14^, ^44^, ^45^ and adds other factors such as dependency on medication, self‐control and the availability of alternative options as factors influencing medication discontinuation. Fear of dependency was a unique factor that influenced participants’ decision to discontinue taking medication. Previous studies have reported that people who use stimulants have a high potential for abuse^46^, ^47^, ^48^; however, there is limited research on whether adults with ADHD are aware of potential dependence on stimulants. Adults’ fear of dependency contributes to their concerns about medication which may result in early discontinuation of medication. Various strategies, such as education about stimulants and the use of non‐stimulants, could be employed to address adults’ concerns about dependency to stimulants and improve adherence.

Self‐regulation was another factor that shaped the needs of participants at both the implementation and discontinuation phase of adherence. Adults’ capacity to self‐control symptoms of ADHD is relatively better than children and adolescents with ADHD.^49^ Our findings suggest that adults may feel better off the medication compared to being on the medication if they can self‐regulate their symptoms. This is particularly important for those whose concerns are higher than their perceived need for medication. Those who can self‐regulate could be trialled for a period without medication; however, the decision to go off medication should be taken in consultation with a physician. This argument is supported by other researchers that suggest discontinuation of medication, particularly if the medication is sub‐optimal in terms of its benefits or if the patient is experiencing severe side‐effects.^17^

The findings of this study reveal factors that are unique to each phase, and factors that are common across the phases. This is parallel to the findings reported by Srimongkon et al^22^ in patients with depression and Vrijens et al^24^ in patients with respiratory problems. Furthermore, the findings of this study are well supported by a study that quantified NCF by using the Beliefs About Medicines Questionnaire (BMQ),^41^ which found that NCF can be effectively used in clinical practice to understand patient's beliefs about medication, predict non‐adherence, and to identify patients’ concerns about medication, which can be used to design tailored interventions to improve adherence. The presence of unique factors highlights the dynamic nature of adherence and suggests that the factors influencing adherence may change along the course of treatment. Therefore, to improve medication adherence, it is important to identify phase‐specific factors, which can form the basis for interventions that are effective and sustainable.

### 4.1 Implications of findings

This study has several clinical and research implications. This study reports the factors that influence medication adherence at the three phases of medication‐taking, which can help physicians in implementing phase‐specific interventions to improve medication adherence in adults with ADHD. This study provides an important insight into the complexity of decision making which includes several compromises and trade‐offs between medication attributes. By recognizing this complexity, physicians can involve adults more in decision making by allowing them to voice their concerns about medication. Knowing patients’ trade‐offs will help physicians in identifying treatment preferences, improving medication adherence and making an informed decision based on patients’ experiences with medication. Furthermore, physicians can better structure clinical consultations by actively listening to patients’ concerns and educating them to address those concerns, which can help in improving adherence and medication outcomes. Future research can quantify the influencing factors at the three phases and examine their relative importance to prioritize interventions.

### 4.2 Limitations

The findings of this study should be interpreted in the context of some limitations. As a qualitative study, the findings are not generalizable to other populations not represented in the study sample. Another potential limitation of this study is the small number of focus groups (n = 3) required to reach saturation; however, this is in line with previously published studies on adherence research.^50^, ^51^, ^52^ Focus groups were used which may have prevented some people from talking about certain issues that they may feel are too private to divulge in front of others. Yet in these cases, the focus groups may have also acted as social support and allowed some participants to divulge more. Although efforts were made to ensure that all participants had an equal opportunity to share their opinions, the possibility of dominance bias cannot be completely ignored.

## 5 CONCLUSIONS

Unique factors were identified that influenced each of the three phases of adherence while some common factors between the phases were also noted. The decision to adhere to medication was based on the balance between adults’ perceived needs and concerns about medication as expected from the NCF. Desire to improve ADHD outcomes was the main motivation for adults to initiate medication. Benefits of medication and its side‐effects were the common factors that influenced the implementation and discontinuation phase of adherence. The negative factors of forgetfulness and medication stigma were unique to the implementation phase while dependency and cost were unique to the discontinuation phase of adherence.

## CONFLICT OF INTEREST

The authors have no conflict of interest to declare.

## Data Availability

The data that support the findings of this study are available on request from the corresponding author. The data are not publicly available due to privacy or ethical restrictions.

## Appendix A COREQ – Consolidated criteria for reporting qualitative studies (COREQ): 32‐item checklist

**Table:**

**Domain 1: Research team and reflexivity**	
*Personal Characteristics*	
1. Interviewer/facilitator *Which author/s conducted the interview or focus group?*	Two focus groups were moderated by PA and one focus group was moderated by MUK.
2. Credentials *What were the researcher's credentials? eg PhD, MD*	PA is a Professor in Medicines Use Optimisation, with the following qualifications: BPharm(Hons), MSc, PhD, Grad Cert Ed Stud (Higher Ed). MUK has following qualifications; MSc, M.Phil. MUK is a PhD student in Pharmacy Practice
3. Occupation *What was their occupation at the time of the study?*	PA is an academic at a public university. She is a Professor in Medicines Use Optimisation. MUK is a PhD student in Pharmacy Practice. Both are pharmacists.
4. Gender *Was the researcher male or female?*	PA is female; MUK is male.
5. Experience and training *What experience or training did the researcher have?*	PA has extensive experience in moderating focus groups. MUK has attended focus groups previously as an observer for taking notes. MUK has also attended a few workshops on ‘qualitative research methods’ during which MUK gained valuable hands‐on training in moderating focus groups.
6. Relationship established *Was a relationship established prior to study commencement?*	Both PA and MUK had no relationship with participants prior to the commencement of the FGs. Prior to commencement of the focus groups, they informed the participants of who they were and what their roles were as part of the study.
7. Participant knowledge of the interviewer *What did the participants know about the researcher? eg personal goals, reasons for doing the research*	On arrival, researchers introduced themselves to the participants and informed them about the purpose of this study.
8. Interviewer characteristics *What characteristics were reported about the interviewer/facilitator? eg Bias, assumptions, reasons and interests in the research topic*	Both MUK and PA have research interests in improving health outcomes in children with ADHD.
9. Methodological orientation and Theory *What methodological orientation was stated to underpin the study? eg grounded theory, discourse analysis, ethnography, phenomenology, content analysis*	The codes were rearranged and grouped based on their relevance to one of the three phases of adherence described in the ‘Ascertaining Barriers to Compliance’ (ABC) framework for examining medication adherence. The Necessity‐Concerns Framework (NCF) was used to explore participants’ needs for and concerns about medication at the three phases of adherence.
10. Sampling *How were participants selected? eg purposive, convenience, consecutive, snowball*	Participants were recruited by a market research company through convenience sampling.
11. Method of approach *How were participants approached?* e*g face‐to‐face, telephone, mail, email*	Participants were recruited by a market research company. The researchers were not involved in the recruitment process.
12. Sample size *How many participants were in the study?*	A total of three focus groups were conducted with twenty participants.
13. Non‐participation *How many people refused to participate or dropped out? Reasons?*	Data were not collected
14. Setting of data collection Where were the data collected? e*g home, clinic, workplace*	Focus groups were conducted at venues intended for group discussions, and within easy access of the participants.
15. Presence of non‐participants *Was anyone else present besides the participants and researchers?*	Only participants and researchers were present in the room where focus groups were being held. No other persons were allowed to enter the room during the discussions.
16. Description of sample *What are the important characteristics of the sample? eg demographic data, date*	Adults (18‐65 years) who were diagnosed with ADHD and prescribed medication for ADHD were eligible to participate. See Table 2 in the manuscript for further information.
17. Interview guide *Were questions, prompts, guides provided by the authors? Was it pilot tested?*	To minimise moderator bias and ensure consistency between the focus groups, a semi‐structured focus group protocol was developed by reviewing previous studies (Hodgkins 2011; Kooij 2013; Semerci 2016; Sobanski 2014; Torgersen 2012) in line with the study objectives. The format and xmlstyle of the questions were in accordance with the criteria proposed by Krueger and Casey (Krueger 2000).
18. Repeat interviews *Were repeat interviews carried out? If yes, how many?*	No repeat focus groups were carried out.
19. Audio/visual recording *Did the research use audio or visual recording to collect the data?*	All focus groups were audio‐recorded only after permission had been provided by the participants.
20. Field notes *Were field notes made during and/or after the interview or focus group?*	MUK a male researcher observed and took field notes in two focus groups while PA was the observer in one focus group discussion.
21. Duration *What was the duration of the interviews or focus group?*	Focus group discussions lasted approximately 60 minutes.
22. Data saturation *Was data saturation discussed?*	No new codes were generated at the 3rd FG, and it was decided that data saturation had been reached after discussion between authors
23. Transcripts returned *Were transcripts returned to participants for comment and/or correction?*	Transcripts were not returned to participants for comment or correction.
**Domain 3: analysis and findings**	
*Data analysis*	
24. Number of data coders *How many data coders coded the data?*	The transcripts were coded by MUK, and the coding discussed with PA.
25. Description of the coding tree *Did authors provide a description of the coding tree?*	The findings have presented under two major themes, in accordance with the Necessity‐Concerns Framework. Within each theme, factors have been categorised based on their influence on the three phases of adherence. The two themes are: Participants’ need for medication Participants’ concerns about medication
26. Derivation of themes *Were themes identified in advance or derived from the data?*	Within each phase, similar codes, derived from the data, were merged to form broader themes.
27. Software *What software, if applicable, was used to manage the data?*	NVivo 11 was used for data management.
28. Participant checking *Did participants provide feedback on the findings?*	No
*Reporting*	
29. Quotations presented *Were participant quotations presented to illustrate the themes/ findings? Was each quotation identified? eg participant number*	Yes, see the Results section of the manuscript.
30. Data and findings consistent *Was there consistency between the data presented and the findings?*	Yes, see the Results section of the manuscript. Participants’ quotes are consistent with the findings.
31. Clarity of major themes *Were major themes clearly presented in the findings?*	The study findings are presented within two broad themes: Participants’ need for medication Participants’ concerns about medication
32. Clarity of minor themes *Is there a description of diverse cases or discussion of minor themes?*	Within each broad theme, findings are discussed in the context of the three phases of adherence (initiation, implementation, discontinuation).

## Appendix B Exploring factors influencing adherence to medication at initiation, implementation and discontinuation of medications in patients with ADHD

### Demographic information of adults

Could you please complete the following questions:

**Age**

(in years) _____________

**Sex**

□ Male
□ Female

**Highest education level**

□ Primary education
□ Secondary education
□ University education
□ Other

**Joint family income (annual, AUD $)**

□ 18 200 or less
□ 18 201‐37 000
□ 37 001‐90 000
□ 90 001‐180 000
□ 180 001 or over

**Marital status**

□ Never married
□ Married
□ Widowed
□ Divorced
□ Separated
□ Other (please specify ………………………………………………………..)

**Country of birth**

□ Australia
□ Other (please specify………………………………………………………….)

**Employment status**

□ Working full‐time
□ Working part‐time
□ Student
□ Not working
□ Other (please specific…………………………………………………………)

Age of at the time of ADHD diagnosis (in years) _______

Please specify the medication you are taking for ADHD (if applicable)

## Appendix C Exploring factors influencing adherence to medication at initiation, implementation and discontinuation of medications in patients with ADHD

### Introduction

Scientific evidence suggests low levels of adherence to medicines by patients with attention‐deficit hyperactivity disorder (referred to as ADHD). These low levels of adherence to medicines can result in poor health outcomes. In order to design interventions to improve adherence to medicines, researchers have to first understand the factors which contribute to non‐adherence. There is limited information currently available about the factors that influence a person's medicine taking, from when a person starts taking their medicine, to their daily medicine taking, and to when they stop taking their medicine.

**Aims**

This study therefore aims to explore what factors affect medicine taking, from starting medicines to when they are stopped, in patients with ADHD.

**Question 1**

Think back to that time when you were first prescribed ADHD medications. How did you feel about starting medications?

**Prompts**

- What were the barriers?
- Name some barriers that first come to your mind and how would you rank them based on their influence?
- What encouraged you to start taking medications?
- Name some facilitators that first come to your mind and can you rank them in order of their influence?

**Question 2**

When did you start your medications?

**Prompts**

- How long did it take you to start your medication after you received the prescription?
- What were your thoughts about starting medications in that period?

**Question 3**

What was your first experience taking those medications?

**Prompts**

- Was it hard to take? Why?
- What made you comfortable taking those medications?

**Question 4**

What made you continue medications after initial use?

**Prompts**

- What were the facilitators?
- What were the barriers?
- Name one each facilitator and barrier that first comes to your mind

**Question 5**

Did you always take the medications as prescribed? Why?

**Prompts**

- Skipping doses intentionally or unintentionally?
- Reducing doses?

**Question 6**

What encourages you to continue your medications as prescribed?

**Prompts**

- Observed medication effects
- Education about disease and medications
- Refill reminder

**Questions 7**

Have you discontinued taking medications? Why?

**Prompts**

- What were the facilitators?
- What were the barriers?
- Name one each facilitator and barrier that first comes to your mind
- Is it for certain period? Why
- Why would you restart medications?

**Question 8**

What can help you to start and continue taking medications as prescribed? Why?

**Prompts**

- What type of professionals you think can help you?
- Do you think healthcare professional can help?
- Which health professional you think can help you the most?
- If that professional contact you periodically, would that be helpful or a nuisance?
- How pharmacist can be helpful in the whole process of medication taking?
